# Non-invasive Auricular Vagus Nerve Stimulation as a Potential Treatment for Covid19-Originated Acute Respiratory Distress Syndrome

**DOI:** 10.3389/fphys.2020.00890

**Published:** 2020-07-28

**Authors:** Eugenijus Kaniusas, Jozsef C. Szeles, Stefan Kampusch, Nuria Alfageme-Lopez, Daniela Yucuma-Conde, Xie Li, Julio Mayol, Christoph Neumayer, Michele Papa, Fivos Panetsos

**Affiliations:** ^1^Faculty of Electrical Engineering and Information Technology, Institute of Electrodynamics, Microwave and Circuit Engineering, Vienna University of Technology, Vienna, Austria; ^2^SzeleSTIM GmbH, Vienna, Austria; ^3^General Hospital of the City of Vienna, Vienna, Austria; ^4^Division of Vascular Surgery, Department of Surgery, Medical University of Vienna, Vienna, Austria; ^5^Faculty of Biology and Faculty of Optics, Complutense University of Madrid, Madrid, Spain; ^6^Department of Clinical Epidemiology and Biostatistics, Pontificia Universidad Javeriana, Bogotá, Colombia; ^7^The Pediatric Department, Women and Children's Hospital of Hunan, Changsha, China; ^8^San Carlos Clinical Hospital, Madrid, Spain; ^9^Institute for Health Research, San Carlos Clinical Hospital (IdISSC), Madrid, Spain; ^10^Faculty of Medicine, Complutense University of Madrid, Madrid, Spain; ^11^Department of Mental and Physical Health and Preventive Medicine, University of Campania “Luigi Vanvitelli”, Naples, Italy

**Keywords:** parasympathetic system, cholinergic anti-inflammatory pathway, hypothalamic pituitary adrenal axis, sympatho-vagal balance, lung inflammation

## Abstract

**Background:** Covid-19 is an infectious disease caused by an invasion of the alveolar epithelial cells by coronavirus 19. The most severe outcome of the disease is the Acute Respiratory Distress Syndrome (ARDS) combined with hypoxemia and cardiovascular damage. ARDS and co-morbidities are associated with inflammatory cytokine storms, sympathetic hyperactivity, and respiratory dysfunction.

**Hypothesis:** In the present paper, we present and justify a novel potential treatment for Covid19-originated ARDS and associated co-morbidities, based on the non-invasive stimulation of the auricular branch of the vagus nerve.

**Methods:** Auricular vagus nerve stimulation activates the parasympathetic system including anti-inflammatory pathways (the cholinergic anti-inflammatory pathway and the hypothalamic pituitary adrenal axis) while regulating the abnormal sympatho-vagal balance and improving respiratory control.

**Results:** Along the paper (1) we expose the role of the parasympathetic system and the vagus nerve in the control of inflammatory processes (2) we formulate our physiological and methodological hypotheses (3) we provide a large body of clinical and preclinical data that support the favorable effects of auricular vagus nerve stimulation in inflammation, sympatho-vagal balance as well as in respiratory and cardiac ailments, and (4) we list the (few) possible collateral effects of the treatment. Finally, we discuss auricular vagus nerve stimulation protective potential, especially in the elderly and co-morbid population with already reduced parasympathetic response.

**Conclusions:** Auricular vagus nerve stimulation is a safe clinical procedure and it could be either an effective treatment for ARDS originated by Covid-19 and similar viruses or a supplementary treatment to actual ARDS therapeutic approaches.

## Introduction

### Covid-19 and Acute Respiratory Distress Syndrome (ARDS)

Covid-19 is an infectious disease caused by SARS-CoV-2 which invades alveolar epithelial cells via angiotensin 2-converting enzyme (ACE2) receptors (Hoffmann et al., 2020; Zheng et al., 2020). Infection is triggered by binding of SARS-CoV-1 or SARS-CoV-2 spike (S) protein to ACE2, which is abundantly expressed in alveolar epithelial cells, thus giving rise to respiratory distress (Vellingiri et al., 2020; Zheng et al., 2020).

Covid-19 may lead—especially in elderly people and/or when comorbidities are present- to the Acute Respiratory Distress Syndrome (ARDS) or fulminant pneumonia. Covid-19 may also lead to severe hypoxemia and increased thrombotic and/or thromboembolic events. As a consequence, acute myocardial injury and even chronic damage to the cardiovascular system may occur since ACE2 receptors are also present in the heart making it susceptible to SARS-CoV-2 virus (Guan et al., 2020; Zheng et al., 2020).

“ARDS is a clinically and biologically heterogeneous disorder associated with many disease processes that injure the lung, culminating in increased non-hydrostatic extravascular lung water, reduced compliance, and severe hypoxemia” (Nanchal and Truwit, 2018). In ARDS, tiny lung blood vessels become leaky, which cause fluid to fill up alveoli and thus to prevent the lungs from effectively providing O_2_ to the rest of the body and clearing CO_2_ (Stevens et al., 2018). ARDS is characterized by widespread inflammation in the lungs, inflammatory cytokine storms, and an imbalance of the sympathetic-parasympathetic activity of the autonomic nervous system (Guo et al., 2020; Zhou et al., 2020).

### The Vagus Nerve and the Immune System

The vagus nerve, the major nerve of the parasympathetic nervous system, mediates and modulates the immune response to inflammatory processes in the body (Pavlov and Tracey, 2012). It is composed by both, sensory (≈80%) and motor fibers (Berthoud and Neuhuber, 2000).

**Figure 1:**
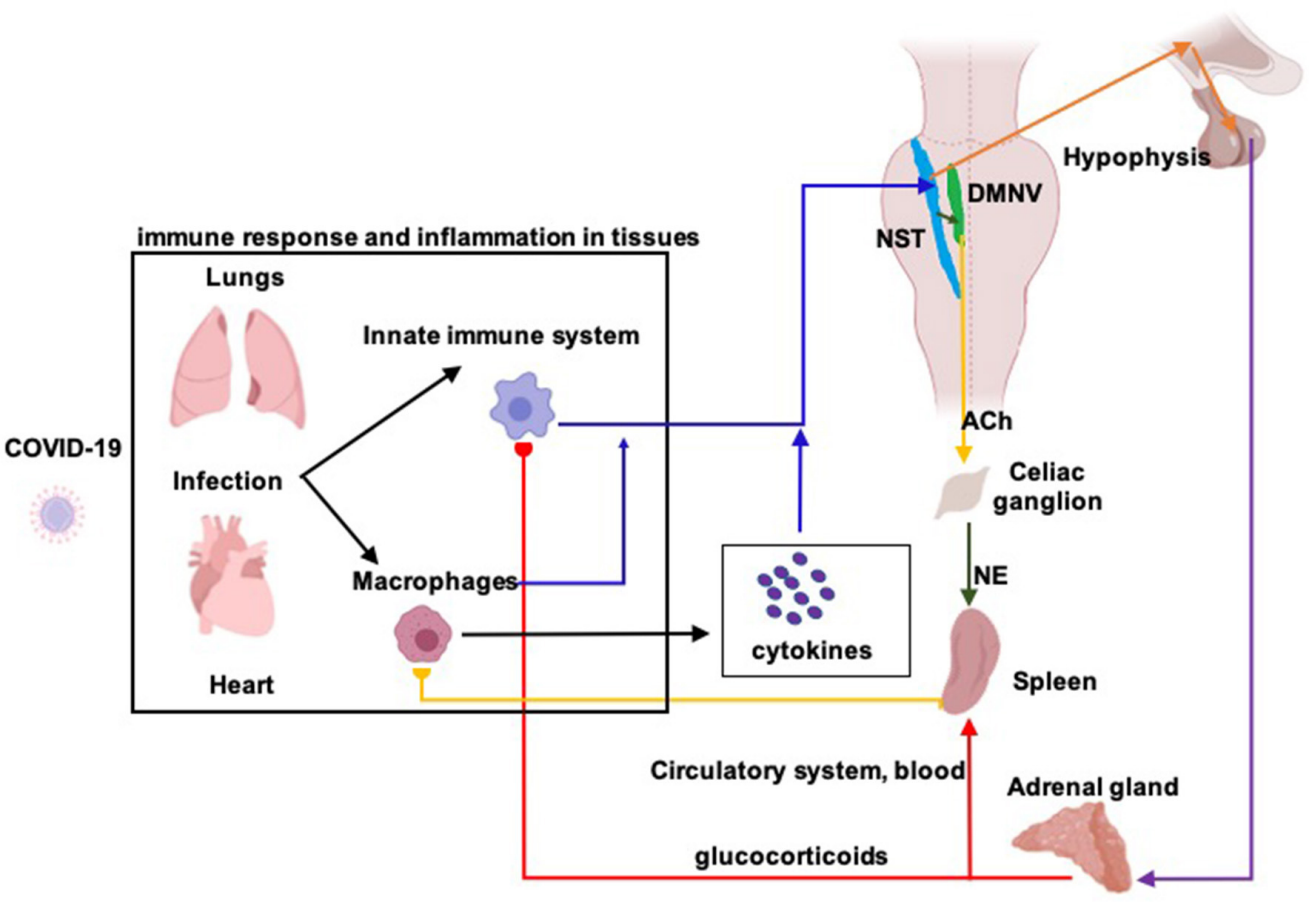
Diagram of the vagus nerve mediated anti-inflammatory responses. The vagus nerve plays a key role in the neuro-endocrine-immune axis, having a dual anti-inflammatory role through its afferent and efferent fibers. In an infection, such as that caused by Covid-19, a primary immune response leads to a release of pro-inflammatory cytokines, generating an inflammatory process at the site of infection, in this case, in the lungs and heart. Released cytokines are recognized by afferent fibers of the vagus nerve (blue arrows; information about inflammation from lung, heart and blood) who transmit such information to the nucleus of the solitary tract (NST). Activation of NST neurons give origin of the anti-inflammatory response which is generated through two different pathways. The first, known as “hypothalamic-pituitary-adrenal axis,” NST efferents to the hypothalamus (orange arrows) stimulate the release of corticotrophin-releasing hormone (CRH) which stimulates the secretion of adrenocorticotropic hormone (ACTH) from the pituitary gland. ACTH reaches the adrenal glands (purple arrow) where stimulates the production of glucocorticoids (cortisol in humans). Glucocorticoids act on the spleen (red arrow), which leads to reduced cytokine release by acting on cells of the immune system. The second, known as the “cholinergic anti-inflammatory reflex,” NST efferents to DMNV, the dorsal motor nucleus of the vagus nerve (black arrow, green nucleus), stimulate the cholinergic motoneurons that project to the splenic nerve in the celiac ganglion (yellow arrow). Acetylcholine (ACh), released from the preganglionic terminals, excites celiac neurons and provoke the release of norepinephrine in the spleen (NE, green arrow). Then, splenic response inhibits macrophages' cytokines release, decreasing inflammation.

Inflammatory mediators (e.g., pro-inflammatory cytokines and/or endotoxins) activate the vagal afferent fibers; the associated afferent fibers convey inflammatory information to the nucleus of the solitary tract (NST) and generate postsynaptic excitatory potentials in NST neurons (Figure 1) (Bonaz et al., 2017). NST somatotopic organization allows the detection and precise localization of any inflammatory process (Pavlov and Tracey, 2012). Once an inflammatory process has been detected, NST neurons activate the dorsal motor nucleus of the vagus nerve (DMNV) whose efferent fibers trigger two different mechanisms of immune reply: the cholinergic anti-inflammatory pathway (ChAP) and the hypothalamic pituitary adrenal axis (HPAA) (Bonaz et al., 2017).

**Figure 2:**
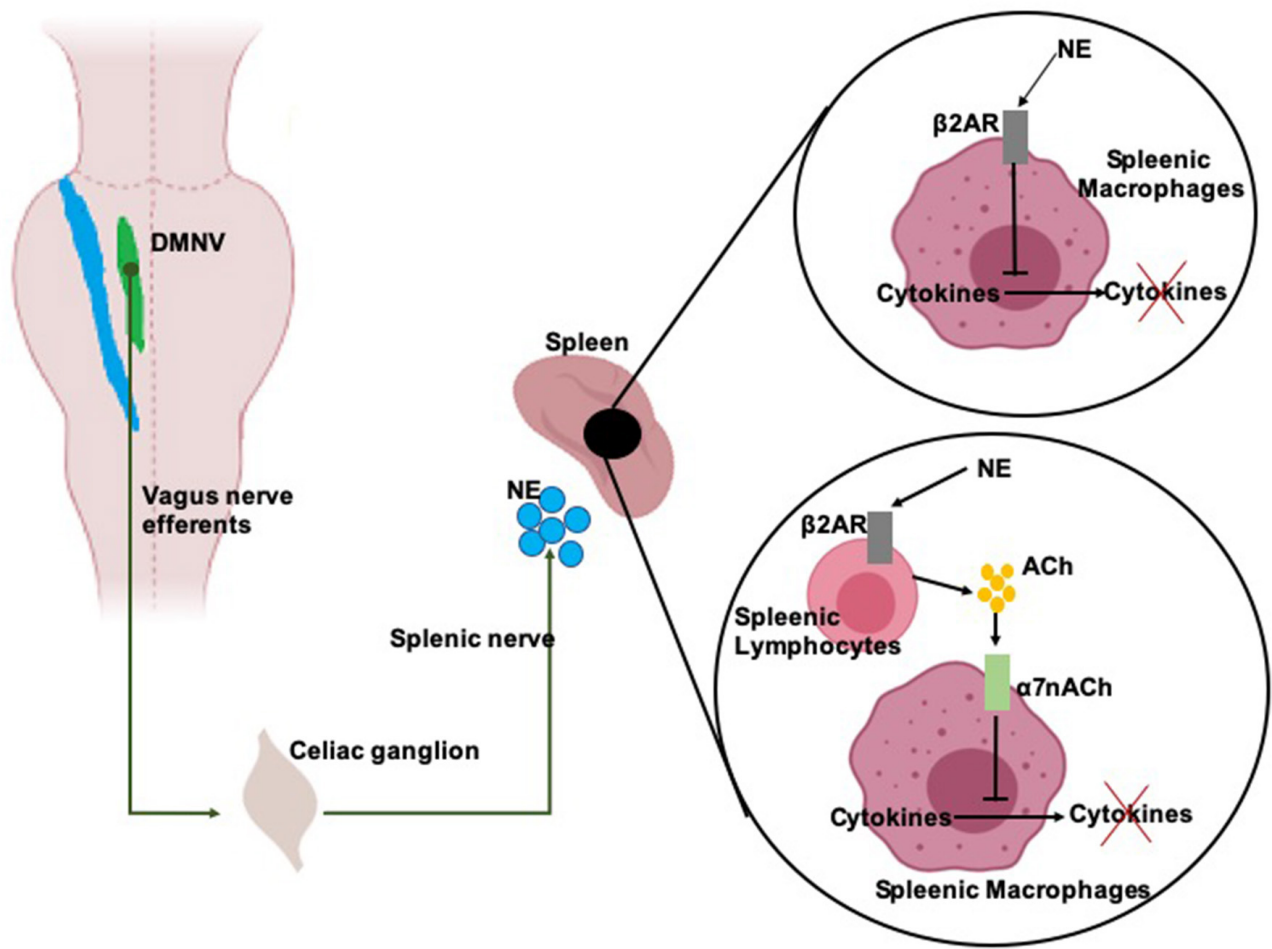
Scheme of the anti-inflammatory activity of the vagal efferents. Vagal efferents arise from the dorsal motor nucleus of the vagus nerve (DMVN) and project to the celiac ganglion, where they synapse with the splenic nerve. DMNV efferents activity provokes stimulates the splenic nerve which release of norepinephrine (NE) over the spleen. NE binds on β2 adrenergic receptors (β2AR) expressed on splenic macrophages and splenic lymphocytes. NE binding on macrophages inhibits the release of pro-inflammatory cytokines of these cells. NE binding on lymphocytes provoke the release of acetylcholine (Ach) which is recognized by α7 acetylcholine receptors (α7nACh) on the membrane of the macrophages. α7nACh activation provokes a disruption of the cytokine release pathway.

ChAP, anti-inflammatory response is triggered by DMNV neurons that project by the adrenergic splenic nerve to the spleen where T-lymphocytes expressing acetylcholine-transferase get activated (Figure 2). When T-cells travel through the body and identify macrophages, they secrete acetylcholine. Acetylcholine binds to acetylcholine surface receptors of macrophages (and/or other cytokine-producing cells), triggering specific intracellular signal transduction to inhibit the release of inflammatory mediators by macrophages (Wang et al., 2003) and thus leading to suppression of pro-inflammatory cytokines (Inoue et al., 2016; Wang et al., 2016). Additionally, infection-activated NST-DMNV neurons provoke an acetylcholine release at their efferents endings, with the aforementioned anti-inflammatory effects (Borovikova et al., 2000). In HPAA, the vagus nerve input to the NST modulates the membrane potential of group A2 noradrenergic neurons which project to the paraventricular hypothalamic area (Figure 1). Parvocellular paraventricular neurons release the corticotropin-stimulating factor which binds to specific receptors expressed by pituitary gland cells. These cells release adreno-corticotropin, a hormone that modulates the cells in the zona fasciculata in the adrenal glands, who release (strongly anti-inflammatory) glucocorticoids in the body (Bonaz et al., 2013).

Precisely, ARDS is characterized by wide inflammatory processes and very high concentrations of cytokines in the lung, just the natural targets of both anti-inflammatory pathways. Interestingly, all ARDS risk factors, e.g., pneumonia, sepsis, gastric content aspiration, trauma, pancreatitis, inhalation injury, non-cardiogenic shock, drug overdose, etc. worsen ARDS symptoms by increasing cytokines concentration and fostering lung inflammation (Umbrello et al., 2017; Yang et al., 2020).

### The Sympatho-Vagal Balance

A homeostasis shift of the autonomic nervous system toward sympathetic predominance (either by severe stress or inflammation) could lead to sympathetic modulation-associated diseases (Stojanovich et al., 2007; Brier et al., 2015). The term “sympatho-vagal balance” denotes the approximate ratio of sympathetic and parasympathetic activity of the autonomic nervous system, frequently given as LF/HF, that means the ratio between low and high frequency components of the power spectrum of the variability of the heart rate, with LF representing both sympathetic and parasympathetic activity and HF representing the parasympathetic activity only (Malik et al., 1996).

In Covid-19, hyperactivity of the sympathetic nervous system may provoke excessive plasma epinephrine and norepinephrine release that lead to pulmonary vasoconstriction and capillary hyperpermeability (see Discussion of Poulat and Couture, 1998; Busl and Bleck, 2015; Liu et al., 2017). At this point an excitatory loop is created in favor to the sympathetic system that provokes an exponential worsening of the symptoms. That is, the acute lung injury provokes a further imbalance in favor to the sympathetic system, with significant plasma IL-6 and IL-10 increases accompanied by considerable hemorrhage, edema, consolidation, atelectasis, neutrophil infiltration, swelling of type I alveolar epithelial and microvascular endothelial cells, mitochondria cavitation, reduction of the mitochondrial crest, vacuolation of the laminar body of type II alveolar epithelial cells, detachment of microvilli, and rarefaction of the basilar membrane (Liu et al., 2017).

Furthermore, the loss of sympatho-vagal equilibrium worsens Covid19-originated inflammation through the renin–angiotensin–aldosterone system, a cascade of vasoactive peptides (Vaduganathan et al., 2020), which has recently been proposed to act as a mediator for ARDS-originated lung injury response (Busse et al., 2020). Indeed, “*the activation of the sympathetic nervous system and the renin–angiotensin–aldosterone system appears inextricably, and reciprocally, linked*,” at least in the case of hypertension patients (Fisher and Paton, 2012). On the other side, ACE2, the receptor for both, SARS-CoV-1 and SARS-CoV-2, who facilitates the endocytosis of the two viruses, is also an activator enzyme of the renin–angiotensin–aldosterone system (Vaduganathan et al., 2020).

## Hypotheses

### Physiological Hypothesis

We hypothesize that (1) a sustained increase of the parasympathetic activity of the autonomous nervous system will activate the ChAP and HPAA anti-inflammatory pathways, which will lead to a strong anti-inflammatory response of the immune system and a consequent containment of the associated ARDS and other diseases; (2) the increase of the parasympathetic activity will invert the abnormal sympatho-vagal balance from sympathetic predominance to parasympathetic one, counteracting the course of ARDS and other diseases.

### Methodological Hypothesis

We hypothesize that the sustained increase of the parasympathetic activity (required for the inversion of the sympatho-vagal balance) can be achieved by a non-invasive electrical vagus nerve stimulation (VNS), namely, by the stimulation of the auricular branch of the vagus nerve (aVNS).

These hypotheses assume that the severity of Covid19-originated ARDS and other diseases can be reduced by an “endogenous systemic brake” which will be naturally given by the parasympathetic part of the autonomous nervous system.

## Methods

### Pathways and Processes

Our hypotheses on the therapeutic effects of VNS-induced parasympathetic activity are supported by a wide range of state-of-the-art clinical and experimental data: decrease of pro-inflammatory cytokines and modulation of lung injuries by activation of the anti-inflammatory pathways, improvement of pulmonary and cardiac functions by adjusting the sympatho-vagal imbalance, etc. (Dos Santos et al., 2011; Krzyzaniak et al., 2011; Kessler et al., 2012; Kox et al., 2012; Levy et al., 2012; Reys et al., 2013; Tarras et al., 2013; Shinlapawittayatorn et al., 2014; Akella and Deshpande, 2015; Chen et al., 2016; Stavrakis and Po, 2016; Liu et al., 2017; Huang et al., 2019).

Electrical stimulation of the vagus nerve can be clinically effective in counteracting Covid-19 complications

- by activating the ChAP,
- by activating the HPAA anti-inflammatory pathway,
- by restoring the sympatho-vagal balance while increasing parasympathetic activity and
  ◦ improving oxygenation
  ◦ favoring modulation of local cardiovascular effects and
  ◦ improving respiratory control.

### Mechanisms by Which Therapeutic Effects of aVNS Will Be Achieved

Covid-19 viral particles invading the lung mucosa, trigger a series of responses of the immune system that lead to the generation of cytokine storms in the body^1^. Covid-19 patients present increased levels of IL-1, IL-2, IL-4, IL-7, IL-10, IL-12, IL-13, IL-17, GCSF, MCSF, IP-10, MCP-1, MIP-1α, HGF, IFN-γ, and TNF-α (Guo et al., 2020). Vagal afferents in the lungs detect inflammatory tissue-damaging stimuli (Carr and Undem, 2003), while VNS reduces the storm of pro-inflammatory cytokines through ChAP and HPAA activation, without leading to immunosuppression (Dos Santos et al., 2011; Krzyzaniak et al., 2011; Akella and Deshpande, 2015; Huang et al., 2019). Consequently, we expect VNS to reduce the severity of Covid-19 inflammation-related complications and fatalities while preventing strong and deteriorating immune responses.

VNS regularizes the sympatho-vagal imbalance and increases parasympathetic activity (Pavlov and Tracey, 2012; Kampusch et al., 2015; Deuchars et al., 2018). We expect it to be especially effective in elderly Covid-19 patients with age-related decline in their natural parasympathetic responses to endogenous and exogenous disturbances and consequent severe complications leading to fatalities. A regularization of the sympatho-vagal balance will decrease the sympathetic activity which in turn will provoke vasodilation and, consequently, improve oxygenation. Additionally, VNS-based release of vasodilating NO (Sator-Katzenschlager et al., 2004; Olshansky et al., 2008; Brack et al., 2009)-combined with VNS anti-inflammatory effects (Chapleau et al., 2016)-mediates cardiovascular responses, thus potentially leading to further improvement of tissues oxygenation (Kaniusas et al., 2015).

Vagus nerve is the major sensory channel from lung/airways to the brain; it controls pulmonary function and it regulates respiration, including normal breathing and respiratory defense mechanisms and provide sensory feedback from the lungs to the brain (Chang et al., 2015). Both, sensory feedback and respiration processes may be significantly impaired by inflammatory conditions: “*It is clear that the function of afferent fibers can be substantively influenced by airway inflammation and remodeling. The perturbations and perversions of afferent nerve function that occur during these states almost certainly contributes to many of the signs and symptoms of inflammatory airway disease*” (Carr and Undem, 2003). In this light, we expect that VNS-provided respiratory feedback will favor lung inflammation control. Furthermore, since the sympathetic-vagal imbalance worsens ARDS symptoms, we expect that VNS balancing function will improve treatment of a wider spectrum of respiratory diseases.

Finally, VNS favorably modulates numerous cardiovascular parameters, yielding to a reduction of blood pressure (Annoni et al., 2015; Mahadi et al., 2019), reduction of arrhythmias (Vanoli et al., 1991; Annoni et al., 2015), and suppression of atrial fibrillation (Stavrakis et al., 2015). VNS blunts sympathetic activity in heart failure (Li et al., 2004; Zhang et al., 2009), and reverses cardiac remodeling after myocardial infarction (Buchholz et al., 2015; Wang et al., 2015), also reviewed in Kaniusas et al. (2019a,b). Thus, VNS could favorably modulate cardiovascular complications in Covid-19 patients, especially in comorbid ones, and reduce the percentage of fatal outcomes.

## Results

### Neurophysiological and Therapeutic Effects of VNS and aVNS

VNS and aVNS have comparable physiological effects (Kaniusas et al., 2019a,b). aVNS-induced cerebral activity patterns alike the activity patterns induced by cervical VNS (Beekwilder and Beems, 2010) and alike favorable healing results (Mercante et al., 2018). aVNS generates far-field brainstem potentials (Berthoud and Neuhuber, 2000; Fallgatter et al., 2003; Polak et al., 2009) were similar to cervical VNS ones (Babygirija et al., 2017; Nonis et al., 2017), while both VNS and aVNS increase brain norepinephrine levels (Lockard et al., 1990; Oleson, 2002; Lehtimäki et al., 2013; Han et al., 2015).

### VNS Therapeutic Effects in ARDS, Lung Injury, and Sepsis

Laboratory research demonstrated VNS lung protective effects (Krzyzaniak et al., 2011). VNS protected against Mesobuthus tamulus venom-induced ARDS in rats, improving parameters like respiratory alterations, hypoxemia, pulmonary edema and histopathological changes (Akella and Deshpande, 2015). It attenuated acute lung injury following burn via the anti-inflammatory pathways (Krzyzaniak et al., 2011) and improved the pulmonary function of acute lung injury while counterbalancing the predominance of the sympathetic nervous system (Liu et al., 2017). VNS was effective to limit pulmonary dendritic cells recruitment within the lung and prevented acute lung injury after burn injury (Lowry et al., 2014).

VNS is a serious candidate for ventilator-induced lung injury, frequently being the consequence of serious lung infections (Johnson and Wilson, 2018). Both, ARDS and acute lung injury, either alone or accompanied by sepsis, can also result in pronounced pulmonary inflammation. Vagus nerve is critical in pulmonary inflammation, whereas vagotomy-induced ChAP interrupt vagotomy leads to a deterioration of the ventilator-induced lung injuries (Dos Santos et al., 2011). Authors also report increased injuries to the alveoli of mechanically-ventilated vagotomized animals as well as hemorrhage and increased levels of IL-6. VNS attenuated the ventilator-induced lung injury by diminishing pro-apoptotic and pro-inflammatory reactions. In lung injury caused by hemorrhagic shock, VNS has been shown to prevent failures of the intestinal barrier and lung injuries (Reys et al., 2013). Interestingly, *in vitro* studies of pulmonary endothelial cells injuries provided evidence that VNS prevents lung injury through activation of the ChAP (Tarras et al., 2013). Furthermore, hemorrhagic shock-caused lung injury can be relieved by VNS which provokes a fall of gut permeability (Levy et al., 2012). VNS showed clear therapeutic effects for hemorrhagic shock mainly due to its anti-inflammatory properties while recovering lung permeability and mitigating lung injury (Powell et al., 2019). Thirty minutes-long VNS trains diminish TNFα and IL-6 the expression in the respiratory brainstem nuclei of developing rats, who control breathing activity of the animal (Johnson et al., 2016). Johnson and Wilson summarize the importance of vagus nerve activity for normal lung function, and the possibility of using VNS to improve outcome in lung injury is very promising (Johnson and Wilson, 2018). Only Kox et al. question the applicability of VNS in systemic and pulmonary inflammation after a two-hit rat model with mechanical ventilation (Kox et al., 2012).

Kessler and co-authors showed in a mice model that interrupt of vagus nerve sensory afferents can deprove the physiological conditions in polymicrobial sepsis (Kessler et al., 2012), while Huang and co-authors showed that VNS mitigates inflammation by re-equilibrating the sympathetic-parasympathetic balance and by reducing the sympathetic activity, with a consequent and thus slowing down the progression of the sepsis (Huang et al., 2010). Patients with vagotomy had no more sepsis or ARDS but more septicemia (Peterson et al., 2012). VNS anti-inflammatory effects counteract lethal endotoxemia or polymicrobial sepsis in mice while reducing systemic TNF (Huston et al., 2007). VNS prevented postoperative ileus and endotoxemia in mice (Hong et al., 2019).

### Therapeutic Effects of aVNS

aVNS effects are systemic: the auricular branch of the vagus nerve projects directly to the NST and through that it excites DMNV neurons that modulate the parasympathetic division of the autonomic nervous system (Figure 1), an in particular the cardiovascular, respiratory and immunological processes. aVNS therapeutic effects (Kaniusas et al., 2019a,b) in inflammation, respiratory dysfunction and cardiovascular diseases include reduction of pro-inflammatory cytokines (Tracey, 2007; Stavrakis et al., 2015), decrease of inflammation level in chronic inflammatory processes like rheumatoid arthritis, postoperative ileus, inflammatory bowel disease (Crohn's disease, ulcerative colitis), systemic inflammation and attenuation of the acute postsurgical inflammatory response after lung lobectomy (Barone et al., 2007; Bernateck et al., 2008; Chakravarthy et al., 2015; Kox et al., 2015; Marshall et al., 2015; Bonaz et al., 2016; Koopman et al., 2016; Kwan et al., 2016; French et al., 2017; Hoover, 2017; Salama et al., 2017). aVNS resulted in an increase of the middle cerebral and supratrochlear artery blood flow velocity in human subjects (Széles and Litscher, 2004) and a decrease of the velocity of the carotid-femoral flow (Hackl et al., 2017). aVNS diminishes systolic blood pressure in subjects with coronary artery diseases as well as in subjects with dysfunctions of glucose metabolism (Zamotrinsky et al., 2001; Huang et al., 2014; Stavrakis et al., 2015) it suppresses atrial fibrillation in patients with paroxysmal atrial fibrillation (Afanasiev et al., 2016) and reliefs anginal symptoms in coronary artery disease patients (Zamotrinsky et al., 1997). aVNS improves cardiac baroreflex sensitivity (Antonino et al., 2017), increases capillary-venous oxygenation in deep tissues in diabetic patients (Kaniusas et al., 2015), increases skin temperature in human subjects with disfunctions of the peripheral arteries and chronic diabetic wounds (Szeles et al., 2013) and improves symptoms in peripheral arterial occlusive diseases (Payrits et al., 2011).

Systemic effects of aVNS are additionally supported by improvement of metabolic processes (Richards and Marley, 1998; Cabýoglu et al., 2006; Huang et al., 2014; Ju et al., 2014; Schukro et al., 2014), attenuation of neurological disorders (Kraus et al., 2007; Hein et al., 2013; Lehtimäki et al., 2013; Kampusch et al., 2015; Bauer et al., 2016; Fang et al., 2016; Rong et al., 2016; Capone et al., 2017; Ylikoski et al., 2017) and enhancement of cognitive performances (Rong et al., 2012; Jacobs et al., 2015) as well as by pain relief effects (Sator-Katzenschlager et al., 2003, 2004; Napadow et al., 2012; Chakravarthy et al., 2015; Kox et al., 2015; Kwan et al., 2016; French et al., 2017; Hoover, 2017; Kovacic et al., 2017; Kaniusas et al., 2019a,b).

### Possible Collateral Effects and Contraindications of aVNS

aVNS can be performed either transcutaneously, in a non-invasive way using surface skin electrodes on the outer ear (Ellrich, 2011; Straube et al., 2015), or percutaneously, in a minimally-invasive way using needle electrodes (Kampusch et al., 2013).

Transcutaneous aVNS employs large surface electrodes which generate diffuse electrical fields that stimulate both, vagal and non-vagal nerve terminals in the ear. Stimulation is safe with minor side effects like headache, dizziness, skin irritation or pain (Badran et al., 2018; Mertens et al., 2018). Stimulation is usually intermittently activated (e.g., NEMOS, Cerbomed GmbH with 3–4 sessions per day, each session lasting at least 1 h) with a total stimulation duration of about 4–5 h per day.

Percutaneous aVNS employs small needle electrodes by which one can achieve a spatially focused electric field that favors precise and specific stimulation of local nerve endings. Electrode contact impedances are lower, allowing an energy-efficient stimulation, with minor side effects (Kaniusas et al., 2019a,b). Skin irritation incidence (<10%) and inadvertent bleeding (<1%) can be reduced to <0.05% if transillumination of the outer ear is employed to visualize auricular vascularization and to prevent erroneous needles placement (Kampusch et al., 2016; Roberts et al., 2016) (Szeles, unpublished clinical data). Also this stimulation is intermittently activated (e.g., AuriStim, SzeleSTIM GmbH with 3 h on and 3 h off). However, it is active during day and night with a much longer effective stimulation duration of 12 h per 24 h than in the case of the transcutaneous aVNS. It is important to underlay that despite such electrodes must be maintained in their place for several days, >80% of the patients expressed very high satisfaction with the cure in terms of quality of life and they reported minor side effects or even total absence of them (Kampusch et al., 2016). The suggested stimulation is 3 h on and 3 h off, with periodic bursts at 1 Hz, and bursts composed out of triphasic patterns with pulse width of 500 μs.

Very light aVNS adverse effects have been reported: Arnold's ear-cough reflex, ear-gag, vaso-vagal reflex, ear-lacrimation, and auriculo-cardiac reflex, all of them being indirect effects of afferent-efferent vagal reflexes, have been observed in only a few percent of the cases (Tekdemir et al., 1998; Ellrich, 2011; Napadow et al., 2012) while headache, syncope, vertigo, alterations of the heart rate and paresthesia have a <1% incidence (Szeles, unpublished clinical data).

aVNS is contraindicated for persons with vagal hypersensitivity, hemophilia, psoriasis vulgaris at application site. aVNS is also contraindicated for patients with active implantable devices, like pacemakers, for their possible interference with the stimulation device.

To our knowledge, there are no reports on special adverse events and contraindications of aVNS in viral infections such as Covid-19.

## Discussion

Based on biophysical principles, we introduce a hypothesis that the severity of Covid-19 complications, especially ARDS, can be reduced by triggering an “endogenous systemic brake” of the inflammatory response. This brake is naturally given by the parasympathetic nervous system and can be accelerated by the electrical stimulation of the vagus nerve.

A large amount of experimental data provides evidence that aVNS activates the parasympathetic anti-inflammatory pathways, equilibrates the sympatho-vagal balance, and improves respiratory and cardiac (Fudim et al., 2020) ailments, making aVNS an excellent candidate for clinical treatment of Covid19-related ARDS and other co-morbidities, including apparently less related diseases, as for example Kawasaki disease, an acute childhood vasculitis of unknown etiology, but associated with viral respiratory infections [infants with classic Kawasaki disease displayed also Covid19-positive symptoms (Jones et al., 2020)].

Furthermore, it has been postulated that the employment of renin–angiotensin–aldosterone system inhibitors could alter ACE2 expression, which in turn could contribute to the expansion of Covid-19 (Diaz, 2020; Fang et al., 2020). The dysregulation of renin–angiotensin–aldosterone system may have detrimental effects on cardiovascular regulation and parasympathetic tone (Fudim et al., 2020), so that aVNS re-equilibrium of the sympatho-vagal balance could possibly counteract these negative effects. Furthermore, hyperactivation of the vagus nerve downregulated the expression or activity of ACE2 which could even potentially prevent viral infection via the ACE2 entry point (Fudim et al., 2020) (see also Osterziel et al., 1994; Duprez et al., 1995; Cody, 1997; Karas et al., 2005; Zucker et al., 2014; Liu et al., 2015).

Last but not least, auricular VNS is an already available, CE-certified, and FDA-approved neuromodulatory therapy for several diseases, safe in application and with absence, or with only minimal, collateral effects.

Physiological data are needed to validate the proposed aVNS therapeutic procedure, to determine the stimulation parameters and to evaluate to which extent it could be used for the treatment of Covid-19 and other virus-originated ARDS. Furthermore, it will be very important to investigate the potential protective functions of aVNS, especially in the high-risk elderly and co-morbid population (Yang et al., 2020). If aVNS is effective, it will help to reduce lung inflammation and, consequently, to reduce the need for hospitalization and for mechanical ventilation, which are the major determinants of healthcare collapse and if fatal outcomes in Covid-19 infection.

Finally, we should investigate if also early aVNS -before ARDS' outbreak- is effective, having a potentially protective function, especially in the elderly and co-morbid population.

## Conclusions

aVNS activates the parasympathetic anti-inflammatory pathways, equilibrates the sympatho-vagal balance, and improves respiratory and cardiac ailments.

aVNS is a safe clinical procedure and it could be an effective treatment for ARDS originated by Covid-19 and similar viruses.

## Data Availability Statement

The original contributions presented in the study are included in the article/supplementary material, further inquiries can be directed to the corresponding author/s.

## Author Contributions

EK and FP contributed to the conceptualization, methodology, investigation, and the writing of the original draft. JS and CN contributed to the supervision. SK, NA-L, DY-C, and XL contributed to the investigation. JM and MP contributed to the writing, review, and editing. All authors contributed to the article and approved the submitted version.

## Conflict of Interest

EK and SK are employed by company SzeleSTIM GmbH. JS received honoraria from SzeleSTIM GmbH and owns patents in the field of the auricular vagus nerve stimulation. EK, SK, and JS are shareholders of SzeleSTIM GmbH. The remaining authors declare that the research was conducted in the absence of any commercial or financial relationships that could be construed as a potential conflict of interest.

## Footnotes

^1^The S proteins of the viral particles of COVID-19 bind to the cells of the pulmonary epithelium by ACE2, fusing with the membrane and releasing their RNA into the cell interior. This RNA, as a pathogen-associated molecular standard (PAMP), is detected by pattern recognition receptors. Generally, they are the Toll-like receptors: TLR3, TLR7, TLR8, and TLR9. Then, RNA is recognized in the cytoplasm by viral RNA receptor I (RIG-I) retinoic acid, gene 5 associated with differentiation of cytosolic receptor melanoma (MDA5), and cyclic nucleotidyltransferase synthase GMP-AMP (cGAS). These signaling adapter complexes, which include the TIR domain-containing adapter protein, including IFN-β (TRIF), mitochondrial antiviral signaling protein (MAVS), and the interferon gene protein stimulator (STING), activate a downstream molecular cascade involving the adapter molecule MyD88. This leads to activation of interferon regulatory factor 3 (IRF3) and of nuclear transcription factor κB (NF-κB), as well as to the production of type I interferons (IFN-α/β) and of several types of inflammatory pro-cytokines.
